# Youth unemployment and mental health: prevalence and associated factors of depression among unemployed young adults in Gedeo zone, Southern Ethiopia

**DOI:** 10.1186/s13033-020-00395-2

**Published:** 2020-08-08

**Authors:** Hirbaye Mokona, Kalkidan Yohannes, Getinet Ayano

**Affiliations:** 1 Department of Psychiatry, College of Medicine and Health Sciences, Dilla University, P.O. Box 419, Dilla, Ethiopia; 2 Department of Psychiatry, School of Medicine, College of Health Sciences, Addis Ababa University, Addis Ababa, Ethiopia; 3 Reserach and training department, Amanuel Mental Specialized Hospital, Addis Ababa, Ethiopia; 4 School of Public Health, Curtin University, Perth, Australia

**Keywords:** Depression, Unemployment, Young adults, Prevalence, Associated factors, Ethiopia

## Abstract

**Background:**

The high rate of unemployment among young adults in Ethiopia, which was 25.3% in 2018, is a major social, and public health concern. The risk of mental health problems like depression is higher among the unemployed than among the employed. However, there was no study conducted on the prevalence and associated factors of depression among unemployed young adults in Ethiopia. Hence, this study was aimed to assess the prevalence and associated factors of depression among unemployed young adults in Gedeo zone, Southern Ethiopia.

**Methods:**

Community based cross sectional study design was employed among 1452 unemployed young adults in Gedeo zone, Southern Ethiopia from May to July, 2019. In order to select the study participants, systematic random sampling technique was used. The presence of depression was assessed by using Patient Health Questionnaire-9 (PHQ-9), and data about socio-demographic characteristics of study participants were collected by using structured questionnaire. Data were coded and entered into Epi-Data version 3.1, and analyzed by SPSS version 20. A multivariable logistic regression analysis was carried out to identify factors associated with depression, and variables with *p* values < 0.05 were considered as statistically significant. The strength of the association was presented by adjusted odds ratio with 95% confidence interval.

**Result:**

The overall prevalence of depression among unemployed young adults in the present study was 30.9% (95% CI: 28.4%, 33.1%). Of the total study participants with depression, 56.7% had mild depression, 36% had moderate depression, and 7.3% had severe depression. Being male (AOR = 1.40, 95% CI: 1.10, 1.80), long duration of unemployment (≥ 1 years) (AOR = 1.56, 95% CI: 1.21, 1.99), low self-esteem (AOR = 1.32, 95% CI: 1.03, 1.68), poor social support (AOR = 1.98, 95% CI: 1.34, 2.93), and current alcohol use (AOR = 1.86, 95% CI: 1.33, 2.59) were significantly associated with depression.

**Conclusion:**

The results of our study indicated that depression is an important public health problem among unemployed young adults in Ethiopia. Therefore, our study suggested that policy makers and program planners should establish appropriate strategy for prevention, early detection and management of depression among this population. Besides, addressing the need of unemployed young people, improving access to care for depression is an important next step. Furthermore, we recommend further studies to understand the nature of depression among unemployed young people, and to strengthen the current results.

## Background

The life period of young adulthood (emerging adulthood) is not only the period of transition from adolescence to adulthood, but also the period of transition from education to employment, which is characterized by high instability [1] and several major life changes such as leaving the parental home, starting a partner relationship, and finding a stable employment [2, 3].

Depressive disorders, as the most common mental problems [4] and leading cause of disability [5], are related to reduced quality of life and increased risk for physical health problems [6]. In 2015, the Global Burden of Diseases study (GBD) estimated that seven of the top 25 causes of Years Lived with Disability (YLD) globally were mental disorders, with major depressive disorder ranked second [7]. Depression among young adults, the period of transition from adolescence to adulthood [8], influences long-term consequences through recurrent depressive episodes [9] and worse socioeconomic outcomes [10] even though it has substantial consequences throughout the lifespan.

According to International Labour Organization (ILO), unemployment is measured using the following 3 criteria; without work, available for work, and seeking work [11]. However, this definition varies in the context of developed and developing countries. In the developed countries where the labour market is largely organized and labour absorption is adequate, unemployment is measured based on the standard definition of the seeking work criteria that is having taken active steps to search for work during specified reference period (i.e. during last 1 week).

On the other hand, in developing countries like Ethiopia, where there is no strong labour market information, labour absorption is inadequate and where the labour force is predominantly self-employed, the standard definition with its emphasis on seeking work criteria is somewhat restrictive and might not fully capture the prevailing employment situation. The relaxed definition which measures unemployment in relation to” without work” and “availability for work” criterion is found to be more plausible in most developing countries.

Employment is a source of financial security, provides people the opportunity to fulfill a social and family role, which is a key prerequisite for both physical and mental health [12]. However, unemployment is a major social problem that determines loss of income, increases the risk of poverty and affect overall health [13, 14]. In addition, unemployment is regarded as a change in social position, particularly a change in family role, and is usually perceived as a very stressful life event [15–17].

The number of unemployed people, in both developed and developing countries, is currently increasing than ever before. Globally, according to International Labour Organization (ILO) report, the number of unemployed people was 192.7 million in 2017, 192.3 million in 2018 and 193.6 million in 2019 [18]. In Africa, based on this report, the number of unemployed people was 37.8 million in 2017, 37.9 million in 2018 and 40.1 million in 2019 [18].

In Ethiopia, according to Ethiopian Central Statistical Agency (CSA), the rate of unemployed people was 16.9% in 2016 and 19.1% in 2018 [19]. The rate of unemployment among young people in Ethiopia was 22% in 2016 and 25.3% in 2018 [19], and these indicated that young people are more affected by unemployment than adults.

Unemployment related elements such as economic or financial distress frequently cause feelings of failure which in turn leads to depression. And also the family and societal pressures associated with job seeking activities and higher expectations from college or university graduates to be employed act as potential mediators of depression among unemployed young adults.

The estimated prevalence of depression among unemployed young adults varies across the studies due to different methods, tools and sample size. A systematic literature review and meta-analysis study found prevalence of depression among unemployed individuals with range from 13 to 14% [20].

Based on the cross-sectional study conducted among 426 unemployed people in United State of America by using the Center for Epidemiological Study Depression Scale (CES-D), the reported prevalence of depression was 29% [21]. According to recent cross-sectional study from Greece conducted among 1064 unemployed young adults by using Depression Anxiety Stress Scale (DASS-21), the reported prevalence of depression was 32.2% [22]. Another cross-sectional study conducted in Spain among 244 unemployed young adults by using Zung’s self-rating depression scale (SDS) showed the prevalence of depression with its severity: 41.8% slight depression, 42.2% moderate depression and 9.3% severe depression [23]. Similar study done in Korea among 124 unemployed young adults by using Beck Depression Inventory-II (BDI-II) found prevalence of depression 39.5% [24]. Another cross-sectional study done in Bangladesh among 304 unemployed young adults by using Depression Anxiety Stress Scale (DASS-21) showed prevalence of depression 49.3% [25].

Several studies have revealed that being male [26, 27], long duration of unemployment [28, 29], low self-esteem [30, 31], poor social support [32–34] and substance use [35, 36] were associated with depression among unemployed young people.

Unemployment among young people has been described as having serious consequences for future lives of young adults and for society at large. Previous studies have suggested that unemployed young people are more likely to have poor physical health [37, 38], engage more frequently in criminal behaviors [39], increased risk of smoking [40], increased risk of alcohol consumption and substance abuse [39, 41]. Moreover, unemployment among young people has been associated with higher mortality rates due to suicide [39, 42] and alcohol-related mortality [43]. Furthermore, unemployment among young adults may increases the risk of psychological crises such as low self esteem, depression, and loss of confidence [44].

Despite World Health Organization (WHO) in 2013 considered unemployed young adults as newly emerged vulnerable groups for mental disorders [45], still there is lack of attention to assess the magnitude of mental health problems among this vulnerable population in African countries, particularly in Ethiopia. To the best of our knowledge no study had been conducted to assess prevalence and associated factors of depression among unemployed young adults in Ethiopia as well as in the study area. Therefore, the present study assessed prevalence and associated factors of depression among unemployed young adults in Gedeo zone, Southern Ethiopia. The findings of this study help health programmers and policy makers at large to design preventive strategies and intervention programs of mental health problems for unemployed young people.

## Method

### Study design and period

Community based cross sectional study was employed to assess prevalence and associated factors of depression among unemployed young adults in Gedeo zone, Southern Ethiopia from May to July, 2019.

### Study area

The study was conducted in the Gedeo zone, Southern Nations, Nationalities and Peoples Region (SNNPR) of Ethiopia. It is located about 375 km south of the capital city, Addis Ababa. The total population of the zone is 1,129,051 persons (565,145 men; 563,906 women) living in 6 districts and 2 town administrations. According to the report from zonal office of women, children, and youth, the unemployment rate among young adults in 2019 was 24.9% (34,724 persons) [46].

### Study populations

All unemployed young adults aged 18–30 years old who were graduated from college or university and living in the study area (in the selected districts and town administration of the zone) for at least 6 months prior to the study were study population. Unemployed young adults who were severely ill and unable to communicate during study period were excluded. In addition, young adults who did not finish schools, or dropped out of college/university were excluded from the study because it is unlikely to be available for work (i.e. being ready for a paid employment) without having educational certificate (i.e. diploma or degree certificate).

### Sample size determination and sampling procedures

In this study, we have tried to calculate sample size for both specific objective 1 (i.e. prevalence of depression) and specific objective 2 (i.e. associated factors of depression), and took the largest sample size. Sample size for specific objective 1 of our study (i.e. prevalence of depression) was calculated by using single proportion formula taking assumptions of: 95% confidence interval, 5% margin of error, and the prevalence of depression among unemployed young adults in Ethiopia is considered to be 50% because per our search we did not find published and even unpublished studies in our country, Ethiopia. Then, we added 10% of non-response rate to the sample size, giving the final sample size of 423. Sample size for specific objective 2 of our study (i.e. associated factors of depression) was calculated using EPI-Info version 7 statistical software (Epi-info/StatCalc) by taking the following assumptions: 80% power, 95% confidence interval, 9.6% prevalence of depression among unemployed males, 15% prevalence of depression among unemployed females and a ratio of 1.66:1 of non-exposed (male gender) to exposed (female gender) which was taken from previous study [47]. We added 10% of non-response rate to the sample size, giving the final sample size of 1452. Therefore, we took the larger sample size for this study (i.e. 1452).

Out of the 6 districts and 2 town administrations of Gedeo zone, 2 districts (Bule district and Gedeb district) and 2 town administrations (Dilla town and Yirgacheffe town) were randomly selected. Then from each selected town administration and town of selected district 3 kebeles (the smallest administrative unit in Ethiopia) were randomly selected again. The lists of unemployed young adults were obtained from Office of Job opportunity creation and food security of each selected district and town administration. According to updated registration from the zonal office of Job opportunity creation and food security, the number of unemployed young adults in the Dilla town administration, Yirgacheffe town administration, Gedeb district, and Bule district were 5483, 3008, 4543, and 4103 respectively. To fix a sampling frame, we conducted census of households with unemployed young adults prior to actual data collection for 1 week by 8 data collectors and numbering of households was done in the selected kebeles. Next, population proportion allocation was done to identify representative study participants from each district and town administration based on the number of the unemployed young adults they have. Then, systematic sampling technique with an interval (K) was used to select study participants. Then after, the first study participant was randomly selected. Finally, every 3 households was interviewed for Dilla town, every four household was interviewed for Gedeb district, every 4 household was interviewed for Bule district and every 6 household was interviewed for Yirgacheffe town. In situations where households had 2 or more eligible study participants, only 1 was randomly selected.

### Data collection tools

A self-constructed structured questionnaire was used to collect data about socio-demographic characteristics of the study participants such as age, sex, marital status, ethnicity, religion, educational level and duration of unemployment.

#### Patient health questionnaire-9 (PHQ-9)

Patient health questionnaire-9 (PHQ-9) based on the DSM-IV criteria was used to assess the presence of depression symptoms with recall period of 2 weeks [48]. The PHQ-9 is a multipurpose instrument for screening, diagnosing, monitoring and measuring the severity of depression. The scale consists of 9-items representing symptoms of depression and each symptom will be rated on a 4-point scale indicating the occurrence and the severity of symptoms: 0 (not at all), 1 (several days), 2 (more than half the days) and 3 (nearly every day). The PHQ-9 items are added up together to give scores ranging from zero to 27. A score of 10 and above indicate presence of depression. A score of 10–14 indicates ‘mild depression’, 15–19 indicates ‘moderate depression, and 20–27 indicates ‘severe depression. The PHQ-9 items showed good internal consistency with Cronbach alpha of 0.799 in the present study.

#### Alcohol, Smoking and Substance Involvement Screening Test (ASSIST)

The presence of substance use was measured by using WHO Alcohol, Smoking and Substance Involvement Screening Test (ASSIST) tool, an 8 item questionnaire developed to assess substance use [49]. The purpose of ASSIST is to detect psychoactive substance use and related problems among primary care patients. It provides information about: the substances people have ever used in their lifetime; the substances they have used in the past 3 months; problems related to substance use; risk of current or future harm; level of dependence; and injecting use. Lifetime substance use is defined as consuming any substances at least once in lifetime and current substance use is defined as use of at least 1 of specified substances for non-medical purpose in the last 3 months [49]. The ASSIST was developed for the World Health Organization (WHO) by an international group of researchers and clinicians as a technical tool to assist with early identification of substance use related health risks and substance use disorders in primary health care, general medical care and other settings [49]. Each question on the ASSIST has a set of responses to choose from, and each response has a numerical score. The Specific Substance Involvement score is calculated by adding together the responses to Questions 2–7 for each of the following locally available substances: tobacco, alcohol, khat (amphetamine type stimulants), and cannabis (marihuana, hashish, ganja). The ASSIST specific substance involvement scores of ≥ 10 for alcohol and ≥ 4 for any substance are an indication of problematic substance use. The ASSIST items showed high internal consistency with Cronbach alpha of 0.946 in the current study.

#### Rosenberg Self-esteem Scale (RSES)

The self-esteem was measured with Rosenberg Self-esteem Scale (RSES) [50]. The RSES determines individual self-worth by measuring both positive and negative feelings about the self. The scale is a 10-item self-report scale designed to measure global self-esteem, the individual’s positive and negative attitude toward the self as a totality. Responses are provided on a 4-point Likert scale ranging from “Strongly Agree” with 3 marks, “Agree” with 2 marks, “Disagree” with 1 mark and “Strongly Disagree” with 0 marks. Items 3, 5, 8, 9 and 10 are reverse scored in which a “Strongly Agree” response attracts 0 mark, “Agree” with 1 mark, “Disagree” with 2 marks and “Strongly Disagree” with 3 marks. The RSES items are added up together to give scores ranging from 0 to 30. A score greater than 15 suggest high self-esteem and scores less than 15 suggest low self-esteem [50]. The Cronbach alpha of RSES in the present study was 0.604.

#### Oslo Social Support Scale-3 (OSS-3)

Social support was measured by using 3 items Oslo Social Support Scale (OSS-3) [51]. The OSS-3 provides a brief measure of social support and functioning, and it is considered to be one of the best predictors of mental health. It covers different fields of social support by measuring the number of people the respondent feels close to, the interest and concern shown by others, and the ease of obtaining practical help from others. In order to score OSS-3, total scores are calculated by adding up the raw scores for each item. The sum of the raw scores has a range from 3–14. The scores of”3–8” indicate poor social support, “9–11″ indicate moderate social support, and “12–14″ indicate strong social support. The Cronbach alpha of OSS-3 in the present study was 0.64.

### Data collection procedures

The questionnaire was first prepared in English; translated to Amharic (local working language) by language experts; and again translated back to English by another person to ensure consistency and accuracy. Next, the data collectors and supervisors were recruited based on previous experience on data collection and supervision. Then, training was given for 3 consecutive days for data collectors and supervisors by the researchers on how to interview, handle ethical issues, supervise and maintain confidentiality and privacy of study subjects. Then after, the data collection instrument was pre-tested on 5% of the actual sample size in similar setting, and amendments were made accordingly. After that, data collection was performed by 8 trained BSc Psychiatry nurses in face-to-face technique (interviewer administered) at the home of study participants, minimizing the risk of misunderstanding questionnaires that may occur in self-administered technique. Data collection was supervised by four MSc Mental health professionals and the principal investigator. Finally, after checked completeness of the required type of data by principal investigator and supervisors, the completed data was coded.

## Data analysis

The supervisors and principal investigator checked the data for completeness, coded and entered into Epi-Data version 3.1 and exported to statistical package for social sciences (SPSS) version 20 for analysis. Means, frequencies, and percentages were used to summarize data and figures, tables and text to present data. Besides, Bivariate analysis was done to describe the associations of each independent variable with depression among unemployed young adults. Variables which had p-value less than 0.2 were considered for the multivariable logistic regression to control the effects of confounding variables. The Hosmer–Lemeshow goodness of fit test was checked for the model. Finally, Variables which had P-values less than 0.05 on multivariable logistic regression were considered as statistically significant and were identified on the basis of odds ratio (OR) with 95% confidence intervals (CI).

## Results

### Socio-demographic characteristics of unemployed young adults

Of 1452 proposed study participants, a total number of 1419 unemployed young adults were included in the present study with the response rate of 97.7% which means 33 (2.3%) refused to participate. The mean age of the study participants was 23.7 (SD ± 3.35) years, and 59% of the participants were in the age range of 18–24 years. Among unemployed young adults participated in the current study, 57.8% were males and 42.2% were females. Of the 1419 respondents, 69.8% were single; 75.1% were Gedeo in ethnicity; and 42.8% were Christian Orthodox in religion. On the other hand, 674 (47.5%) of participants had diploma educational level; and 48.5% of participants reported poor social support. Regarding duration of unemployment, 67% of study participants had duration of unemployment less than 1 year and followed by those who had ≥ 1 year duration of unemployment, 33%. Of the total study subjects participated, 55.5% reported high self-esteem, while 44.5% reported low self-esteem, (Table 1).

**Table 1:** Socio demographic characteristics of unemployed young adults in Gedeo zone, Southern, Ethiopia, 2019 (N = 1419)

Variables	Frequency	Percentage
Age in years( mean = 23.7, SD = ± 3.35)		
18–24	837	59%
25–30	582	41%
Sex		
Male	820	57.8%
Female	599	42.2%
Marital status		
Married	428	30.2%
Single/divorced/separated	991	69.8%
Ethnicity of study participant		
Gedeo	1066	75.1%
Others^a^	353	24.9%
Religion		
Orthodox	607	42.8%
Protestant	597	42.1%
Muslim	167	11.7%
Others^b^	48	3.4%
Educational level		
Certificate	605	42.6%
Diploma	674	47.5%
Degree and above	140	9.9%
Duration of unemployment		
< 1 year	951	67%
≥ 1 year	468	33%
Social support		
Poor social support	688	48.5%
Moderate social support	545	38.4%
Strong social support	186	13.1%
Self-esteem (Mean = 15.58, SD ± 3.55)		
Low self-esteem	631	44.5
High self-esteem	788	55.5

^a^Oromo, Amhara, Gurage, Wolaita

^b^Catholic, Adventist

### Prevalence of depression among unemployed young adults

**Fig. 1:**
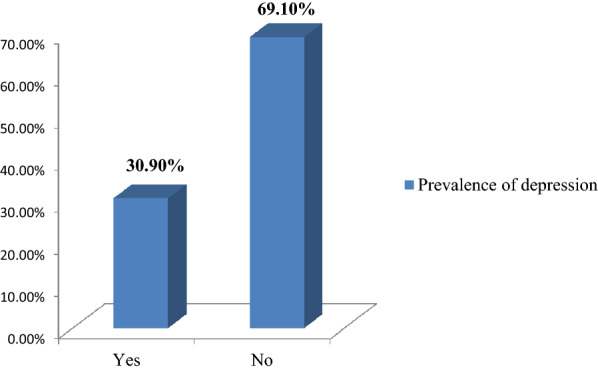
Prevalence of depression among unemployed young adults living in Gedeo zone, Southern Ethiopia, 2019

**Fig. 2:**
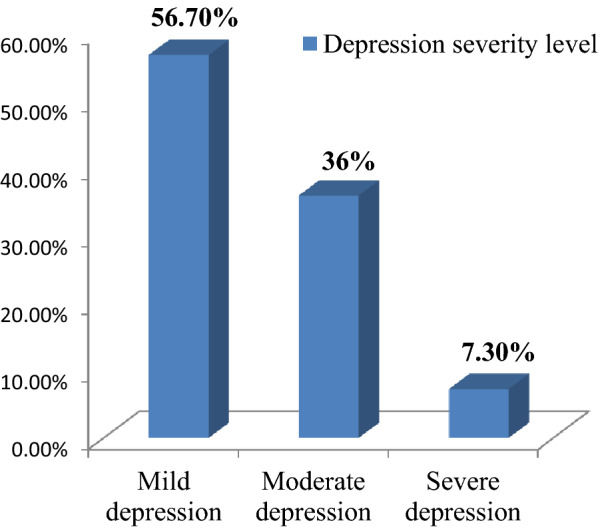
Depression severity level among unemployed young adults living in Gedeo zone, Southern Ethiopia, 2019

As indicated on the Fig. 1, the overall prevalence of depression among unemployed young adults in the present study was 30.9 (95% CI: 28.4, 33.1%). Of the total unemployed young adults with depression, 56.7% had mild depression, 36% had moderate depression, and 7.3% had severe depression (Fig. 2).

### Substance use among unemployed young adults

**Fig. 3:**
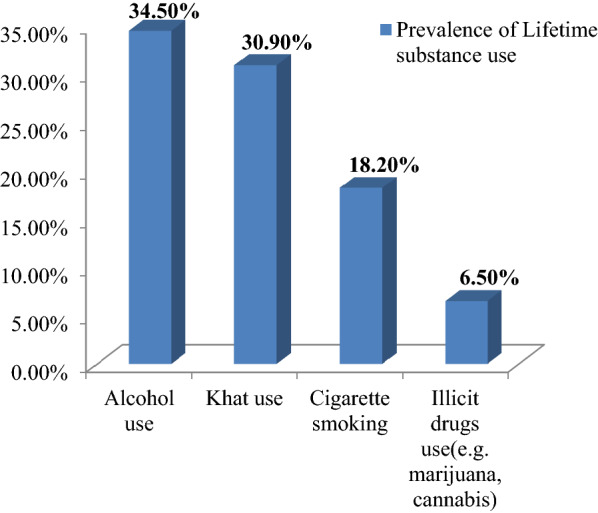
Prevalence of lifetime substance use by type of substance among unemployed young adults living in Gedeo zone, Southern Ethiopia, 2019

Both lifetime and current substance use was measured in our study by using ASSIST WHO tool. The overall prevalence of lifetime substance in our study was 44.7%. Of the total lifetime substance users; 34.5% were alcohol users, 30.9% were khat users (Khat-Amphetamine type stimulant), 18.2% were cigarette smokers, and 6.5% were illicit drug users (e.g. marijuana, cannabis) (Fig. 3).

**Fig. 4:**
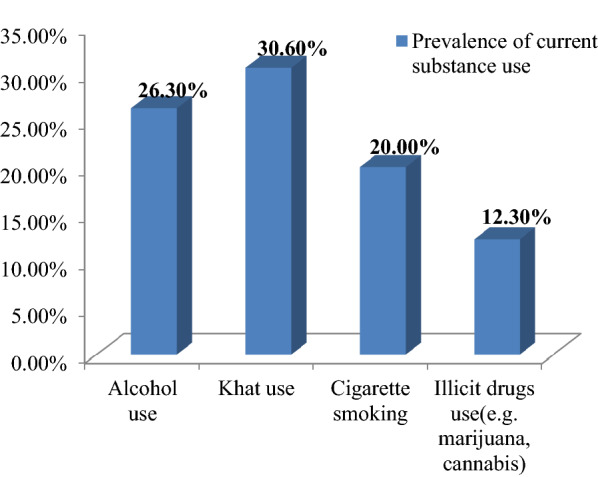
Prevalence of current substance use by type of substance among unemployed young adults living in Gedeo zone, Southern Ethiopia, 2019

On the other hand, the overall prevalence of current substance use in the present study was 38.8%. Of the total current substance users, 26.3% were alcohol users, 30.6% were khat users, 20% were cigarette smokers, and 12.3% were illicit drug users (e.g. marijuana, cannabis) (Fig. 4).

### Factors associated with depression among unemployed young adults

During the bivariate logistic regression analysis, variables such as age (25–30 years), sex (being male), long duration of unemployment, low self-esteem, poor social support, current cigarette smoking, current alcohol use, current khat use, current illicit drug use (e.g. marijuana, cannabis) were associated with depression (had p-value less than 0.2) and entered into multivariate logistic regression analysis for further analysis. On the other hand, variables such as educational level and marital status were not associated with depression and therefore excluded from further analyses.

In the multivariable logistic regression analysis, variables such as sex (being male), long duration of unemployment, low self-esteem, poor social support, and current alcohol use were statistically significant with depression among unemployed young adults, while there was no statistical difference between unemployed young adults with depression and those without depression, with respect to age, current cigarette smoking, current khat use, and current illicit drug (e.g. marijuana, cannabis) use.

Hence, unemployed young men were at higher risk for depression as compared to unemployed young women (AOR = 1.40, 95% CI: 1.10, 1.80). The likelihood of depression among unemployed young adults with long duration of unemployment (≥ 1 year) was found to be 1.56 times as compared to those with short duration of unemployment (< 1 year). Unemployed young adults with low self-esteem were at higher risk for depression (AOR = 1.32, 95% CI: 1.03, 1.68) as compared to those with high self-esteem. Unemployed young adults with poor social support were 1.98 times at risk for depression as compared to those with strong social support. The likelihood of depression among unemployed young adults with current alcohol use was found to be 1.86 times higher as compared to those without current alcohol use (Table 2).

**Table 2:** Factors associated with depression among unemployed young adults living in Gedeo zone, Southern Ethiopia, 2019 (N = 1419)

Variables	Depression		COR (95% CI)	AOR (95% CI)
	No	Yes		
Age in years				
18–24	604	233	1	1
25–30	376	206	1.42 (1.13–1.78)*	1.17 (0.91–1.49)
Sex				
Male	532	288	1.61 (1.27–2.03)*	1.40 (1.10–1.80)*
Female	448	151	1	1
Duration of unemployment				
< 1 year	705	246	1	1
≥ 1 year	275	193	2.01 (1.59–2.54)*	1.56 (1.21–1.99)*
Self-esteem				
High self-esteem	581	207	1	1
Low self-esteem	399	232	1.62 (1.30–2.05)*	1.32 (1.03–1.68)*
Social support				
Poor social support	425	262	2.11 (1.45–3.08)*	1.98 (1.34–2.93)*
Moderate social support	411	135	1.13 (0.76–1.67)	1.05 (0.69–1.58)
Strong social support	144	42	1	1
Current cigarette smoking				
No	823	313	1	1
Yes	157	126	2.11 (1.61–2.76)*	1.21 (0.85–1.73)
Current alcohol use				
No	778	268	1	1
Yes	202	171	2.46 (1.92–3.14)*	1.86 (1.33–2.59)*
Current khat use				
No	714	271	1	1
Yes	266	168	1.66 (1.31–2.11)*	0.98 (0.71–1.33)
Current marijuana/cannabis use				
No	873	372	1	1
Yes	107	67	1.47 (1.06–2.04)*	0.90 (0.61–1.34)

*Statistically significant (p-value < 0.05)

1 = Reference variable

## Discussion

Our study revealed a high prevalence of depression in sample of unemployed young adults residing in Gedeo zone, Southern Ethiopia. To the best of our knowledge, this is the first community-based cross-sectional study that has investigated the prevalence and associated factors of depression among unemployed young people aged 18–30 years in Ethiopia. The prevalence of depression among unemployed young adults in the current study was 30.9%. Our finding was consistent with the findings of previous studies conducted in Greece 32.2% [22] and in USA 29% [21]. However, the prevalence of depression among unemployed young adults in the present study is significantly higher than the finding of systematic literature review and meta-analysis study conducted by Paul and Moser [20] that found the prevalence range of depression from 13 to 14% among unemployed individuals.

On the other hand, the finding of our study is lower than the finding of study done in Germany among 365 long-term unemployed individuals by using Hospital Anxiety and Depression Scale (HADS) which was 37% [52]. Another study conducted in Germany also reported the higher prevalence of depression (34.4%) among long-term unemployed people measured by using Patient Health Questionnaire-9 (PHQ-9) as compared to the result of our study (30.9%). Also the results of studies conducted in Spain (51.5%) [23], Korea (39.5%) [24] and Bangladesh (49.3%) [25] were higher than the finding of our study.

The reason for variation might be due to difference in sample size. The assessment instrument might also be the possible reason for the differences in the prevalence of depression among unemployed people. For instance, the previous studies conducted in Germany, Greece, and Bangladesh used Depression Anxiety Stress Scale (DASS-21) to assess depression, whereas our study used the PHQ-9. The other explanation for the difference might be due to the type of data collection procedure that researchers used (interviewer administered versus self-administered) and the study settings (community based versus institutional based).

The present study identified factors associated with depression among unemployed young adults. Sex was identified as significant variable, as unemployed young men were at higher risk for depression as compared to women. Our study finding is consistent with findings of previous studies that found unemployed men were more likely to be affected by depression [26, 27], but the finding of our study is inconsistent with those of previous studies that reported unemployed women exhibited higher rate of depression [28, 47, 52]. The reasons why unemployed men are more affected by depression than women might be explained by: first, unemployed men experience stronger distress as traditional values and social expectations make employment more important for men- unemployment might be experienced by men as a personal failure. Second, masculine identity is intricately linked to having a job in most developing countries including Ethiopia and is severely threatened by unemployment related loss of income that threatens other areas of goal attainment, including capacities to provide security for self and family, which leads to depression. Third, for women, on the other hand, work is seen as only 1 of several roles (e.g. the role of being wife and mother are assumed to be as important as work in women’s lives). For example, Dew and Bromet [26] found that unemployment has a lesser impact on women which might be due to different gender roles with women valuing their jobs less and gaining more self-esteem from their family. Additionally, 2 main arguments based on the study conducted by Shamir [53] to explain the reason why depression is more common among unemployed men than women were; First, men are assumed to have a higher commitment to the work role than women, resulting in stronger distress when deprived of this role. Second, women are assumed to have an alternative role that can serve as a substitute to employment.

In our study, we found that the likelihood of depression among unemployed young adults with long duration of unemployment (≥ 1 year) was 1.56 times more likely as compared to those with short duration of unemployment (< 1 year). Our finding is consistent with findings of previous studies [28, 29] that support the link between unemployment duration and poor mental health: the longer a person is unemployed, the worse mental health outcomes (e.g. depression). The possible explanation could be due the fact that unemployed young adults experience continued and more and more discouraging failures in job seeking and financial pressures that become stronger as time passes.

In the present study, we found that unemployed young adults with low self-esteem were at higher risk for depression as compared to those with high self-esteem. This finding is supported by the findings of previous longitudinal studies conducted in United State of America [30, 31]. Several pathways have been proposed that explain why people with lower self-esteem might be at higher risk for depression. For example, according to Beck’s cognitive theory of depression, negative beliefs about the self, which are central to low self-esteem, would contribute to the development of depressive disorders [54].

Unemployed young adults with poor social support were 1.98 times at risk for depression as compared to those with strong social support, which is in agreement with the findings of previous studies that found poor social support was strongly correlated with depression among unemployed young people [32–34]. There has been previous study that indicates social support has an important influence on the mental health of unemployed people. For instance, a population based case–control study on young people conducted in Sweden found that mental health was generally poor among unemployed persons with low social support from family and friends than among unemployed persons with higher social support [55].

Our study revealed that the likelihood of depression among unemployed young adults who reported current alcohol use was found to be 1.86 times higher as compared to those who did not report current alcohol use. The finding of our study is supported by the evidences from 2 cohort studies conducted by Fergusson et.al [35, 36] who found alcohol abuse /dependence was most likely to lead to depression. The possible reason is that alcohol might be used as a self-medication strategy against distress or unemployment-related struggles- a way to deal with financial hardship, which in turn increases likelihood of depression in this population.

Several strengths of this study need to be highlighted. First, our study is the first study to assess prevalence of depression and associated factors among unemployed young adults in Ethiopia, which can be considered as strength. Second, our large sample which was recruited from Community sample with an excellent response rate can also be considered as strength. Third, we used standard tool that takes a non-judgmental and more acceptable approach to measure depression and other variables. On the other hand, this study has several limitations. First, our study used a cross-sectional study design that makes it difficult to determine the causality of the observed associations between depression and its associated factors. Second, due to the sensitive nature of the study in terms of social stigma, the study participants may have underreported depression and some other variables such as substance use. Third, the exclusion of young adults who did not finish school, or dropped out of college/university may have resulted in an underrepresentation of severe cases of depression. Fourth, the study did not include detailed risk factors (i.e. income or financial distress, coping strategy, and emotional intelligence) which might contribute for depression among this vulnerable population.

## Conclusions

The results of our study indicated that depression is an important public health problem among unemployed young adults in Gedeo zone, southern Ethiopia. Being male, long duration of unemployment, low self-esteem, poor social support, and current alcohol use were statistically significant with depression. Therefore, our study suggested that Policy makers and program planners should establish appropriate strategy for prevention, early detection and management of depression among unemployed young adults. Besides, it is important to design and implement effective community based depression prevention programs for unemployed young adults in Ethiopia. Furthermore, addressing the need of unemployed young people, improving access to care for mental health problems, particularly for depression, is an important next step. Moreover, we recommend further studies to understand the nature of depression among unemployed young people, investigating other contributing factors by using different study designs to strengthen the current results.

## Data Availability

Due to ethical issues and protection of confidentiality of the study participants, raw data cannot be provided. But, the summary data are available in the main document. When needed they are available from the corresponding author on reasonable request.

## Abbreviations

ASSIST: Alcohol, Smoking and Substance Involvement Screening Test
DASS-21: 21-item Depression Anxiety Stress Scale
CI: Confidence interval
ILO: International Labor Organization
OR: Odds ratio
OSS-3: Oslo 3-item Social Support Scale
PHQ-9: Patient Health Questionnaire-9
RSES: Rosenberg Self-Esteem Scale
SNNPR: South Nations, Nationalities and Peoples’ Region
SPSS: Statistical Packages for Social Sciences
WHO: World Health Organization
